# Ignorance and Superstition Factors in the Treatment of School Children

**Published:** 1912-09-15

**Authors:** Jacob Sobel

**Affiliations:** New York. Borough Chief, Division of Child Hygiene, Department of Health, New York City


### IGNORANCE AND SUPERSTITION FACTORS IN THE TREATMENT OF SCHOOL CHILDREN.

JACOB SOBEL, M.D., NEW YORK.

Borough Chief, Division of Child Hygiene, Department of Health, New York City.

Picture a massive beautiful building, palatial in its gray stone architecture and planned from the white tiled basement to the roof garden that overlooks leagues of city and sea front to accommodate and make comfortable a thousand or so little alien public school children. The quaint street scenes that surround this school attest to its conglomerate child army.

Here, in the street that faces the school, a score of Old World merchants display on push carts their stores of ruddy apples, shining tinware and gaudy wearing apparel. There a mother of Italy, wearing the costume of her native land, leads a little brown-eyed tardy boy to the school gates.

Even the drone of human voices that fills the streets is a mixture of Irish, Bohemian, Yiddish, Italian, Hungarian, Polish, French, Chinese, Scandinavian—every tongue but English. As we listen the hum of voices deepens.

These mothers of the East Side who have been summoned at the request of the health authorities to give their permission for remedial health measures which have been advised are questioning among themselves the wisdom of these measures. New York city is trying to bring about physical efficiency as well as mental efficiency in its army of school children, but the wall of superstition which encloses the foreign born parent and almost shuts out the ministration of science is voiced in the scraps of gossip which reach our ears.

"If the tonsils are taken out of my child's throat it will be too wide, the air will rush into the lungs too quickly and produce inflammation of the chest," one says, with a note of authority in her tone.

"The Lord made my child as he done make me, and I ain't going to have her changed," cries a negro mother.

From another mother comes this statement: "I don't want the doctor to fix Sammy's ears. A running ear is good for him and cleans out his system. Anyhow, he hears too much at home as it is."

The burden of all the gossip is the same. It is an operation that these mothers fear. There was a time when to mention the word operation meant a hurry call for the police and only the dismissal of the entire school would appease the angry mob of mothers. Such an incident is rare now, but the superstition of our foreign element is a factor always to be measured in the effort which the Department of Health is making to bring about greater efficiency in the children of the tenements.

Whether the crusader for physical welfare be the school doctor, the school nurse, the district nurse or the visiting physician who goes into the homes of our alien neighbors to attempt to overcome the continual violation of the laws of health that prevails, this health officer is met with the same fusilade of superstition and distrust. He must tear down a fortress of prejudice, tradition, doubt, ignorance, irresponsibility and apprehension before he is able to make any headway with health. Meeting this ignorance of the foreign born parent is a more difficult problem today than that of combating disease.

There was the case of Annie S., a squinting, bow-legged little girl of the East Side who was discovered by the local health officer, while inspecting her class in school, to be sadly in need of glasses. She was backward in her studies not because of any lack of mentality, but because she was unable to see for more than a foot before her nose.

The Department of Health wanted to correct Annie's defective vision, but the idea was scoffed at in the child's home. Annie's mother met the suggestion that her little girl wear glasses with a host of excellent reasons why she should not. She said that eyeglasses were a luxury worn only for "style" and they would "get Annie so used to them that she wouldn't be able to see without them." She would be subjected to ridicule at the hands of her playmates, a frequent and favorite taunt being "Oh, you four eyes!"

Then came the climax of all these objections to Annie's eye saving spectacles. The mother of the East Side said that the wearing of them would interfere seriously with the matrimonial prospects of her ten-year-old daughter. "She won't marry well because of them," her mother reiterated. "If Annie needs glasses let her husband buy them," she finished with decision.

A further investigation into the cause of Annie's defective vision disclosed the fact that when she was a baby her inflamed eyes had been made far worse by the injection into them of breast milk. The glasses were procured, however, after much persuasion on the part of a tactful visiting nurse, and Annie not only caught up with her classmates in school but eventually far outstripped them.

There was Allie Z., too, a child who seemed to be almost feeble minded, but whose only physical defect was enlarged tonsils and nasal obstruction caused by an exaggerated case of adenoids. These defects had impaired the proper ingress of air to Allie's lungs, had disturbed his sleep, causing restlessness and night terrors, had interfered with his hearing so that he appeared stupid in school, had resulted in a deformity of the jaw, making speech and voice defective, and had even stunted his growth and development. The department of Health wished to provide free hospital care for Allie that the adenoid growth might be removed and the boy be given a fighting chance at health, but the child's parents met the idea with an array of superstitions.

His grandmother suggested that Allie's "palate was down" and followed this enlightening statement with the advice that by constant pulling of the hair on top of the child's scalp the palate would be elevated and the condition relieved. His mother stated definitely when told of the enlarged, diseased condition of the child's tonsils, "Is that so? Sure, the Lord put them there and there they'll stay." And his father was positive that the removal of adenoids gave a child suicidal tendencies. These prejudices were overcome, however, and the child was wonderfully relieved and helped by his operation.

Voodooism, the superstition of the negro, finds its victims in this city as well as in the Southern States. I have come in personal relation with cases where the use of different colored yarn was prescribed by the "doctor" for the cure of contagious and other diseases found in school children; red yarn for erysipelas, yellow for jaundice, pink for "pink eye" and white for anaemia. An educated negro, when advised to have his adenoids removed, indignantly replied, "The negro is in a great measure characterized by his flat nose and yet you advocate the removal of my adenoids, which would result in making my nose higher. I shall never do that. Always be what you are."

One inspector, when working in a school district frequented by negroes, was much perplexed at the stubbornness of a large number of cases of ringworm, only to learn that the best treatment for this disease was held to be the application of a round piece of cloth which the voodoo doctor carefully applied, mumbling diligently the while.

A familiar picture of the tenements is the Italian bambino, big eyed baby, wrapped as tightly as any Egyptian mummy in round upon round of tightlys tretched swaddling bands. Sometimes the arms are included in this wrapping; always the little limbs are so tightly bound that the baby can only lie upon a mattress upon the floor or be stood up against the wall or any other convenient resting place.

Argue with the baby's mother against the wearing of these swaddling clothes which bind the baby, impede its freedom of motion and interfere with his breathing and circulation and you are told that this mummylike garb will keep the legs straight and the feet small. Like the Chinese the Italians believe that small feet are a distinct advantage for girls. Carmella, the sister of the bambino, is dressed in an array of garments of many sizes, shapes and colors which may be pulled off one after the other, like coats of an onion, and are supposed to protect her from the dangers of cold and the discomforts of low temperature.

The Italian parent also believes that the unsightly crust of eczema so often seen on the scalp of infants is a protective covering placed there by an all wise Providence. To remove it, the mother believes, will cause the death ot her child. Even a suggestion that olive oil be applied—and olive oil according to the Italian mind is surely good for what ails you—is not over enthusiastically received and the appearance of an otherwise highly entrancing little Angelina or Raphaelo is marred.

If the visiting nurse suggests that Benny's hair, which is matted together by dirt or scalp disease, be cut off the mother's horror is exemplified in this statement: the child's strength will go with it. If the nurse advises that the baby's nails be cut short she is told that this will make him a thief or retard his speech. Bite them off, yes; cut them off, never!

Fancy entering a home on the lower East Side for the purpose of instructing a mother in the care of the mouth and teeth to be shown her toothless jaw and then to be told in a significant jargon "I lost my teeth too and I'm alive." Every negress will tell you that pulling teeth gives children sore eyes and that it is bad luck for any poor child to wear gold or silver in its mouth.

### "EVIL EYE" PLAYS ITS PART.

One mammy said, "I pull my chile's teeth and they is mighty lucky if they kin get the holes stopped up with meat and bread." Woe betide you if you chance to step over the little one as it plays on the floor in his home. Doesn't this retard his growth? You must atone for your mistake, retrace your steps and recross it.

If you say nice things about the healthy little chap on the floor, admire it and try to be friendly with it, its mother will hastily lick its eyes and face three times. How else can she remove the "evil eye" which you have unknowingly cast upon it? If she does not resort to this procedure it is only because she has scared away this evil spirit by sewing some salt in the child's shirt and tying a red ribbon around its wrist or neck.

So the bugbear of ignorance and superstition takes its stalking way in the wake of us doctors. Why do you insist upon telling the mother that she must not give the child a "taste of everything?" Doesn't this indiscriminate tasting harden the child? And doesn't deprivation at this time mean that in after years the child will suffer from want, hunger and unsatisfied desires? You are told that a child has ringworm because he plays in the circles which children so frequently chalk for games on the sidewalk.

You say that the childs backwardness in his school studies is due to some physical defect, only to be looked at in wonder by the mother and told that the child's memory is weak because he persists in eating the ends of the bread loaves. Anaemia and malnutrition are so common in the public school children of this city that from 20,000 to 25,000 of them are seriously handicapped. This means improper housing, poor ventilation, a lack of personal cleanliness and the need of food of proper quality, quantity, selection, preparation and palatability. But many mothers will tell you that their children are pale because they look into the looking glass late at night.

STOLEN MEAT AS A DOCTOR.

I recently had occasion to advise a mother as to the existence of adenoid growths and pigeon breast in her child with this result. Before my next visit the child was taken to the coffin of a religious individual and the latter's hand was rubbed several times over the child's pigeon breast, when presto, the deformity was supposed to decrease. After advising proper measures for the removal of warts and moles, I have been told that a small piece of meat stolen from a butcher and buried in the earth will cause the growth to shrivel and disappear, simultaneously with the disintegration of the buried meat.

I remember too, one little Italian youngster who was always shy when I approached him, afraid, it seemed to me, as if some one were about to do *him* bodily harm. Subsequently I learned that having red hair he was looked upon as "bad" and as such was beaten regularly—a therapeutic measure advised by his mother.

Progressive medicine has taught us that at the very onset of an attack of diarrhoea in infancy and childhood and during its entire course, milk in every form must be discontinued. A large proportion of the Hebrew and Italian element, while accepting this advice as applicable to bottled milk, condensed milk, loose milk, or the various milk foods, cannot and will not see its application to breast milk.

They reason that inasmuch as mother's milk is the ideal food and as the child is becoming weakened by the discharges of the disease, breast milk should be given and if anything a little more frequently than before, failing to realize that in doing so they are virtually adding fuel to an intestinal fire and diminishing very materially the infants' prospects of recovery. Water, always so necessary for infants and children, particularly during the heated term, is often denied them because it is supposed to create worms.

The Italian mother whose child is suffering from poor nutrition is with great difficulty persuaded to discontinue giving it wine, beer and coffee. These are considered tonics and are used to strengthen the children. Many of these traditions and superstitions have been handed down from father to son, from family to family and from neighbor to neighbor and have so taken hold of our foreign population as to be actual beliefs and convictions.

Their danger lies not so much in the fact that they do actual harm—for many of them are harmless—but that in combating them much valuable time is lost by the health officer during which proper measures which make for health might be instituted. Meeting the hoodoo of the tenement means the exercise of tact and judgment, patience and perseverance, kindness and encouragement, enthusiasm and hope. These qualities we are packing in our medicine cases daily, together with our instruments and vials and by means of them we are reclaiming Annie, Allie and the rest to health and every other efficiency.—*New York Times.*

